# Differential Inhibition of Signal Peptide Peptidase Family Members by Established γ-Secretase Inhibitors

**DOI:** 10.1371/journal.pone.0128619

**Published:** 2015-06-05

**Authors:** Yong Ran, Gabriela Z. Ladd, Carolina Ceballos-Diaz, Joo In Jung, Doron Greenbaum, Kevin M. Felsenstein, Todd E. Golde

**Affiliations:** 1 Department of Neuroscience, Center for Translational Research in Neurodegenerative Disease, and McKnight Brain Institute, College of Medicine University of Florida, Gainesville, Florida, United States of America; 2 College of Pharmacy, University of Florida, Gainesville, Florida, United States of America; 3 Pennsylvania Drug Discovery Institute, Philadelphia, Pennsylvania, United States of America; Nathan Kline Institute and New York University School of Medicine, UNITED STATES

## Abstract

The signal peptide peptidases (SPPs) are biomedically important proteases implicated as therapeutic targets for hepatitis C (human SPP, (hSPP)), plasmodium (*Plasmodium* SPP (pSPP)), and B-cell immunomodulation and neoplasia (signal peptide peptidase like 2a, (SPPL2a)). To date, no drug-like, selective inhibitors have been reported. We use a recombinant substrate based on the amino-terminus of BRI2 fused to amyloid β 1-25 (Aβ_1-25_) (FBA) to develop facile, cost-effective SPP/SPPL protease assays. Co-transfection of expression plasmids expressing the FBA substrate with SPP/SPPLs were conducted to evaluate cleavage, which was monitored by ELISA, Western Blot and immunoprecipitation/MALDI-TOF Mass spectrometry (IP/MS). No cleavage is detected in the absence of SPP/SPPL overexpression. Multiple γ-secretase inhibitors (GSIs) and (*Z-LL*)_2_ ketone differentially inhibited SPP/SPPL activity; for example, IC_50_ of LY-411,575 varied from 51±79 nM (on SPPL2a) to 5499±122 nM (on SPPL2b), while Compound E showed inhibition only on hSPP with IC_50_ of 1465±93 nM. Data generated were predictive of effects observed for endogenous SPPL2a cleavage of CD74 in a murine B-Cell line. Thus, it is possible to differentially inhibit SPP family members. These SPP/SPPL cleavage assays will expedite the search for selective inhibitors. The data also reinforce similarities between SPP family member cleavage and cleavage catalyzed by γ-secretase.

## Introduction

In 2002, a novel family of proteins similar to presenilins (PSENs) was identified through bioinformatic and biochemical methods [1–3]. The newly discovered proteins were eventually named signal peptide peptidases (SPPs) and signal peptide peptidase-like proteins (SPPLs). Multiple proteins have since been identified as substrates of SPP/SPPLs [4, 5]. Highly conserved through evolution, SPP/SPPLs are present in organisms from human to plants and appear to mediate a wide array of biological function. Four of the five human SPP/SPPLs (hSPP, SPPL2a, SPPL2b, SPPL2c and SPPL3) have different subcellular localization, which likely plays a role in reported differences in substrate cleavage preference [4]. The first identified member of the SPP family and sharing the name, SPP, is known to play a role in immunosurveillance [6, 7], virus maturation [8–11], protein dislocation [12] and normal development [13, 14]. SPPL3 is the closest homolog of SPP and has been reported to cleave transfected SPP substrates and the foamy virus envelope protein, and like SPP, plays a role in embryonic development [15, 16]. SPPL2a, SPPL2b and SPPL2c share greater similarity than other SPP family members [5]. To date SPPL2c has not been detected at the protein level and no substrate has been reported. SPPL2c gene lacks introns and is highly polymorphic suggesting it may be a pseudogene, however of the 171 coding variants identified from 6503 human exomes, only 2 are due to a frameshift, which is atypical for most pseudogenes (Exome Variant Server, NHLBI GO Exome Sequencing Project (ESP), Seattle, WA (URL: http://evs.gs.washington.edu/EVS/)). SPPL2a and SPPL2b are not only highly homologous, but also share cleavage substrates. Of the six SPPL2a/b substrates reported, four are shared by SPPL2a and 2b: TNF-α [17, 18], BRI2 [19, 20], CD74 [21–23] and Transferrin receptor 1 (TfR1)[24]. Given the high diversity of their substrates and the obvious similarities to hSPP, SPPL2a/b are likely to have numerous biological functions. One essential function is the role of SPPL2a in B cell development. The invariant chain (li, CD74) of the major histocompatability class II complex (MHCII) undergoes intramembrane proteolysis mediated by SPPL2a [21–23]. Depletion of SPPL2a leads to accumulation of an NH_2_-terminal fragment (NTF) of CD74 which impairs B cell development and survival. Although SPPL2b can cleave CD74 when overexpressed, it does not appear contribute to CD74 NTF turnover [25].

The study of PSENs and SPPs has been linked based on their structural and functional similarities. Both PSs and SPPs share the same YD and GxGD active site motifs. Predicted transmembrane topologies also indicated the hypothesized catalytic aspartate residues of each protein are present within adjacent and opposing transmembrane regions, a finding confirmed by structural data obtained from x-ray crystallography of a SPP family member [1–3, 26]. SPPs and γ-secretase preferentially catalyze the cleavage of transmembrane proteins that either have short extracellular/lumenal domains, or have been previously cleaved in a process referred to as ectodomain shedding [27, 28]. In the case of γ-secretase, this ectodomain shedding is mainly attributed to α- and β-secretase activities [29]. For SPP, the classical signal peptidase cleavage performs this“shedding” [2, 30], and for SPPL family members, it appears that some substrates can be cleaved by metalloprotease disintegrins (ADAMs) [16–18, 20]. Another interesting finding indicates that human SPPL3 itself might be the first GxGD-type aspartyl protease to be capable of acting as a sheddase when processing a foamy virus envelope protein mutant (FVenv)[31]. Despite the similarity between SPPs and PSs, predicted topology suggests that the orientation of all human SPPs is inverted relative to PSs. This feature likely accounts for why PSENs preferentially recognize and cleave Type I transmembrane proteins while SPPs prefer Type II transmembrane protein with some exceptions [31–34]. Another major difference between PSENs and SPPs is that PSENs require three cofactor proteins, Aph-1, Pen-2, and Nicastrin in a complex to properly function as a protease[27, 35], while transient heterologous expression of SPP, SPPL2a, 2b or SPPL3 alone is sufficient to observe proteolytic activity. This suggests that SPPs either does not need cofactors or that other unknown cofactor(s) are widely present in mammalian cells [2].

The intense focus on PSENs as a therapeutic target in Alzheimer's disease resulted in the development of extremely potent γ-secretase inhibitors (GSIs) targeting both the active site aspartates and allosteric sites of PSENs. Recent studies using γ-secretase inhibitors provide indirect evidence in support of the hypothesis that PSENs and SPPs share similar catalytic mechanisms. Some GSIs, e.g., LY-411,575 and L-685,458 inhibit the activity of SPP [36–38], indicating SPP and PSs share a common binding site. Differences in topology may also influence responses to certain inhibitors. The GSI DAPT has been found to selectively inhibit PSENs, but not SPPs despite a structure similar to LY-411,575 [38]. The peptide-based SPP inhibitor, (Z-LL)_2_ ketone does not inhibit γ-secretase cleavage of amyloid precursor protein (APP), but acts like an inverse γ-secretase modulator (iGSM) by altering processivity of γ-secretase cleavage [39, 40].

**Fig 1:**
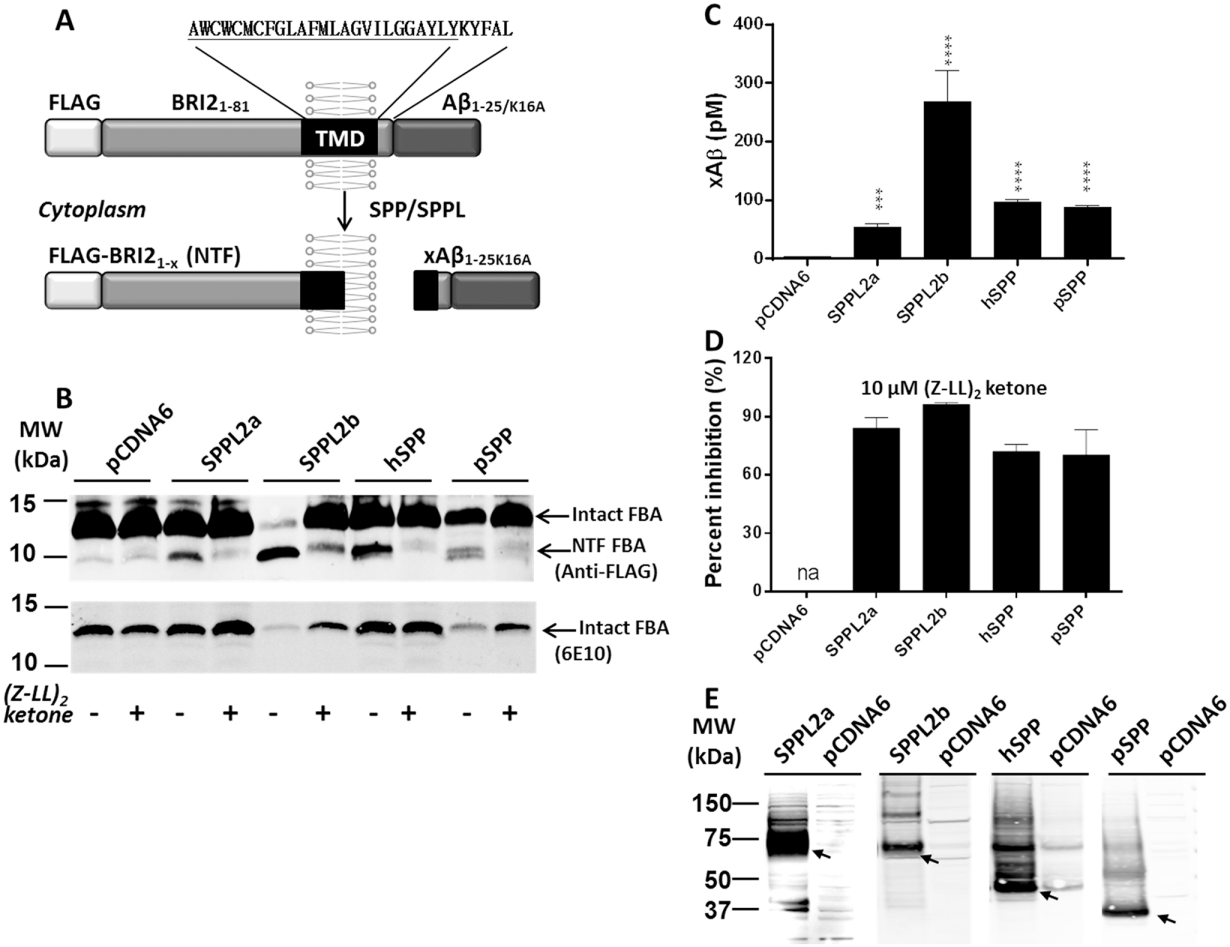
The FBA substrate is efficiently cleaved by SPP/SPPLs. A. Design of SPP assay substrate based on BRI2 transmembrane domain. A FLAG tag fused to the NH_2_-terminus of BRI2_1-81_. Aβ_1-25/K16A_ fused to the COOH-terminus of BRI2_1-81_. Potential cleavage in the transmembrane domain releases the ectodomain fragment xAβ_1-25/K16A_. B. Western blotting of FBA ±SPPL transfected cell lysates with and without 10 μM (Z-LL)_2_ ketone. Blot detected with anti-FLAG M2 antibody. The intact FBA and ICDs are marked with arrows. C. SPP/SPPLs significantly increase xAβ_1-25/K16A_ secretion from FBA transfected cells. xAβ_1-25/K16A_ levels were determined with Aβ ELISA. D. The FBA cleavages conducted by SPPLs co-transfection are largely inhibited by 10 μM (Z-LL)_2_ ketone. E. Western blotting of SPPLs transiently transfected HEK cell lysates. Blots detected with antisera against SPPL2a, SPPL2b, hSPP, and pSPP respectively. Monomer bands are marked with arrows. All experiments repeated 3 times. Statistical analysis performed by 1-way ANOVA ((*p<0.05, **p<0.01, ***p<0.001, ****p<0.0001).

In order to fully evaluate the potential of therapeutic utility of targeting SPP family members, it is essential to develop drug-like inhibitors highly selective to the various members of the family. Currently, there is no rapid inhibitor screening method available for SPP/SPPLs. Most current assays for SPP/SPPLs rely on Western blotting to assess substrate cleavage, which is not well suited for HTS. Further, we have had challenges in miniaturizing our luciferase based reporter system for SPPs and extending these assays to report on SPPL cleavage [36]. In this report, we designed a recombinant substrate based on NH_2_-terminus of BRI2 (ITM2B) protein and amyloid β 1–25 (Aβ_1–25_) peptide (referred to as FBA, see Fig 1a). The NH_2_-terminus of BRI2 includes the intracellular domain and transmembrane domain, which includes a potential cleavage site of SPPLs. A few residues on the ectodomain side remain to preserve the integrity of the putative transmembrane topology, while avoiding a potential sheddase cleavage site. Aβ_1–25_ was chosen here because: i) it is a natural secretion peptide in the process of APP; ii) it is stable in cell culture media[39] and iii) sensitive and cost-effective enzyme-linked immunosorbent assay (ELISA) method is available. Using this substrate, we established a facile proteolytic activity assay for SPP, SPPL2a, SPPL2b and pSPP. All four proteases cleave the FBA substrate, but are differentially inhibited by the only reported SPP specific inhibitor (Z-LL)_2_ ketone and five different GSIs. We also document that these proteases prefer different cleavage sites on the same substrate and that the cleavage, akin to γ-secretase, may be processive. The development of this rapid screening tool will facilitate future efforts to identify selective drug-like inhibitors of these proteases.

## Materials and Methods

### DNA and cell culture

Expression constructs in pcDNA6 for COOH-terminal FLAG (DYKDDDDK) and strep (WSHPQFEK) tagged SPPL2a, SPPL2b, hSPP and pSPP were generated as previously described [15, 36, 41, 42]. Untagged versions of all enzymes were also made and ligated into the pAG3 vector. Substrates (Fig 1) were designed as follows: FLAG-BRI2_1-81_- Aβ_1-25/K16A_ (FBA). The K16A mutation was incorporated to prevent potential α-secretase cleavage that would preclude ELISA based detection of the released COOH-terminal fragment. The substrate DNA was cloned into the expression vector pcDNA6. Constructs were confirmed by DNA sequencing. The overexpression of substrates was performed by transiently transfecting human embryonic kidney (HEK 293T) cells with or without SPP/SPPL vectors. Cells were grown in DMEM media supplemented with Hyclone 10% fetal bovine serum (GE, Logan, Utah, USA) and 1% penicillin/streptomycin (Life Technologies, Grand Island, NY, USA). Briefly, 2.7 μg DNA was applied to a 75% confluent 6 well plate (Corning, Tewksbury, MA, USA) using the polycation polyethylenimine (PEI) transfection method. Inhibitor testing experiments were conducted in 48 well plates with appropriately adjusted amounts of DNA and PEI and incubated 12–16 hours, after which the growth medium was replaced with fresh medium. DMSO (Sigma, St. Louis, MO, USA) and GSIs were added at the appropriate concentration. The GSIs, L685,458 and Compound W were purchased from Tocris (Tocris, Bristol, UK). (Z-LL)_2_ ketone, Compound E, DAPT, GSI II were purchased from Calbiochem (Merck Millipore, Billerica, MA, USA). LY-411,575 was synthesized by A. Fauq at the Mayo Clinic Chemical Core. Twenty-four hours later, the medium was collected for assay by ELISA and MS. A20 cells (TIB-208) (ATCC, Manassas, VA, USA) were cultured in RPMI-1640 medium (ATCC) supplied with 10% fetal bovine serum, 1% penicillin/streptomycin and 0.05 mM 2-mercaptoethanol (Life Technologies). At the density of 5x10^5^ cells/ml, A20 cells were spun down at 1000 g for 2 min and resuspended in fresh medium supplied with DMSO or GSIs and incubated for 16 hr.

### ELISA and Western Blotting

Sandwich ELISAs used for Aβ detection were performed as previously described [39]. Briefly, Aβ and Aβ adducts with substrate TMD in conditioned media was captured with Ab5 antibody (human Aβ_1–16_ specific) and detected with horseradish peroxidase mAb 4G8 (Covance, Princeton, NJ, USA). Synthetic Aβ_1–40_ was used as standard. All ELISAs were developed with TMB substrate (KPL, Gaithersburg, Maryland, USA). Bis-Tris precast gels (Biorad, Hercules, CA, USA) were used for all SDS-PAGE. Monoclonal anti-FLAG M2 antibody (Sigma) and Aβ_1–16_ antibody 6E10 (Covance) were used for Western blotting. Antisera against SPPL2a, SPPL2b, hSPP and pSPP were generated as previously described [42] and were used at dilution of 1:1000 for Western blotting. Anti-CD74 In-1 antibody (BD Bioscience, San Jose, CA, USA) was used for A20 cell lysate Western blotting at dilution of 1:1000.

### Immunoprecipitation and mass spectrometry

Immunoprecipitation and mass spectrometry of xAβ_1–25_ in conditioned media were performed as previously described [39]. In this study, 10 ml conditioned media were used for each experiment. ICDs were immunoprecipitated with anti-FLAG M2 magnetic beads. Cells from 10 cm dished treated with DMSO or 20 μM LY-411,575 were used for each test.

## Results

### FBA is a cleavable substrate for SPP/SPPLs

An expression plasmid encoding the FBA substrate (Fig 1A) was transiently transfected into HEK 293T cells and expression was confirmed by Western blotting (Fig 1B). Intact FBA protein appeared at ~13 kDa and is detected by anti-FLAG M2 and anti-Aβ_1–16_ 6E10 antibodies. The FLAG M2 antibody detected an additional, yet faint band at ~10 kDa, which may represent a minor cleavage product of FBA. However, in the absence of SPP/SPPLs co-expression, 10 uM of (Z-LL)_2_ ketone treatment did not reduce levels of the 10 kDa band indicating this is not likely the result of an SPP/SPPLs mediated cleavage. Given that signal peptidase might produce a similar sized intracellular domain (ICD), this “constitutive” 10 kDa ICD might represent the inefficient cleavage of FBA by signal peptidase. When detected with 6E10, only the main band at 13 kD was detected (Fig 1B bottom panel). Co-transfection of FBA with SPP/SPPLs resulted in lower levels of the ~13 kD FBA substrate and the appearance of a more intense band at ~10 kD (Fig 1B top panel). The SPP/SPPLs-dependent 10 kD band was only detected by anti-FLAG M2 antibody, suggesting the COOH-terminal Aβ epitope was cleaved from the FBA substrate and the ICD of FBA remained in the cells. The effects of each SPP/SPPL on the FBA substrate were different, despite the fact that equal amounts of DNA were transfected. SPPL2b reduced levels of the intact FBA substrate by >90%, while the other three enzymes reduced levels of intact FBA to a much lesser extent. The multiple ICDs from pSPP co-transfection cells suggest pSPP may have more than one cleavage site (Fig 1B). The addition of 10 μM (Z-LL)_2_ ketone significantly reduced the generation of the SPP/SPPL dependent 10 kDa ICD and increased levels of the intact FBA substrate when co-expressed with the SPP/SPPLs.

SPP/SPPLs dependent cleavage of FBA was further confirmed by Aβ ELISA using conditioned media. COOH-terminal fragment (CTF, xAβ_1-25/K16A_) levels in conditioned media from FBA transfected cells did not reach the detection limit (~5 pM) of the Aβ ELISA (Fig 1C). Co-transfection of FBA with SPPLs increased Aβ levels to 50–400 pM depending on the SPP/SPPLs species and cell culture conditions. In support of the corresponding Western blot data, SPPL2b is the most active—yielding 200–400 pM Aβ in the ELISA. Over 75% inhibition of FBA cleavage for each SPP/SPPL was observed when 10 μM (Z-LL)_2_ ketone was applied to cells overnight (Fig 1D). To evaluate the effects of COOH-terminal tags on each enzyme, untagged SPPLs were also co-transfected with FBA. Appropriate CTFs were created for each enzyme (data not shown). Given the congruence between the Western blot and ELISA results, these data demonstrate that i) FBA is cleaved by SPP/SPPLs with an ICD detected in the cell-lysate and ii) the CTF of FBA is efficiently secreted into the media.

Over expression of SPPL2a, SPPL2b, hSPP and pSPP was confirmed by Western Blotting using antisera for each SPPL. The monomeric form was the dominant species observed, with lesser amounts of dimer and trimer (Fig 1E) via SDS-PAGE analysis [42]. Apparent molecular weights of SPPL2a, SPPL2b and hSPP roughly matched their corresponding predicted molecular weights of 63, 69, and 48 kD, respectively. The pSPP monomer produced a band at 37 kD instead of calculated weight of 52 kD. With the exception of pSPP, faint bands with a similar migration pattern to hSPP, SPPL2A and SPPL2a were detected in the control samples, which might be the endogenous enzymes.

### Each SPP/SPPL has preferential cleavage sites on FBA

**Fig 2:**
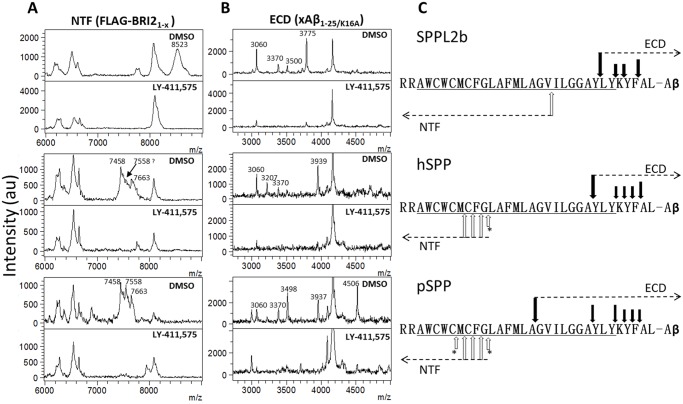
Each SPP/SPPL has preferential cleavage site on FBA. A. Cell lysates of FBA-SPP/SPPL co-transfected cells are used for IP/MS with anti-FLAG M2 magnetic beads. Top and bottom panels are the spectra for cells with DMSO or 20 μM LY-411,575. Main peaks observed are labeled with molecular weight (Da). The peaks below 7000 Da and at ~8080 Da are non-specific. B. 10–20 ml of FBA/SPP co-transfected cell culture media is used for IP/MS with the anti-Aβ_1–16_ antibody AB5 bound to magnetic beads. Main peaks observed in the xAβ_1-25/K16A_ (ECDs) are labeled with molecular weights. The peaks at ~4190 Da are non-specific. C. Schematic representation of the cleavages of FBA. Putative TMD is underlined. Open and solid arrows indicate observed ICDs and xAβ_1-25/K16A_ respectively. “Minor” peaks labeled with * are not shown in these spectra, but have been observed in others (See S1 Fig).

The intramembrane cleavage sites of TNF-α and TfR 1 by SPPL2b are the only substrates that have been characterized in cells [18, 24]. hSPP processing of viral core protein signal peptides and synthetic prolactin signal peptide have also been reported [43–45]. We were able to evaluate substrate processing of each SPPL using the same substrate. The FLAG tagged ICDs and secreted CTF FBA ectodomains (xAβ_1-25/K16A_, ECDs) produced from SPPL2b, hSPP and pSPP co-transfected cells were immunoprecipitated using anti-FLAG M2 and anti-Aβ_1–16_ AB5 antibodies. Representative MALDI-TOF spectra are shown in Fig 2. Despite considerable effort, we could not identify the ICDs or CTFs produced by co-transfection of SPPL2a and FBA, likely due to low cleavage efficiency. A broad peak at 8523 Da was observed in FBA/SPPL2b co-transfected cell lysate, but not in the 20 μM LY-411,575 treated cells. This peak matches the calculated MW of FLAG-BRI_1-V68_. A series of peaks ranging from 7325 Da to 7803 Da was observed in the FBA/hSPP and FBA/pSPP co-transfected cell lysates, but not in LY-411,575 treated cells (Table 1). Within this range, only the 7663 Da peak matches the calculated MW of FLAG-BRI_1-F59_. The other peaks match single or double acetylated FLAG-BRI ICDs from FLAG-BRI_1-G60_ to FLAG-BRI_1-C56_. Consistent with the IP/MS data, we could detect lysine acetylation on the FBA substrate using an acetylated lysine specific antibody (see Panels A and B in S1 Fig). All ICDs ended inside the putative TMD of FBA. The multiple cleavage sites utilized by pSPP may explain the multiple bands shown in Western blot (Fig 1B). The major xAβ_1–25_ species produced in SPPL2b transfected cell media are BRI_L75_-Aβ_1-25/K16A_ and BRI_A80_-Aβ_1-25/K16A,_ with BRI_K77_ and BRI_Y78_-Aβ_1-25/K16A_ as minor products. BRI_Y74_-Aβ_1-25/K16A_ and BRI_A80_-Aβ_1-25/K16A_ are the dominant products of the hSPP transfected cell media. The major products in the pSPP cell media are BRI_G67_, BRI_Y74_ and BRI_K77_-Aβ_1-25/K16A_. All cleavage products detected in the media are listed in Table 2. No major cleavage product was detected in conditioned media treated with 20 μM LY-411,575, which suggests all detected cleavage products are SPP/SPPL dependent. Each SPP/SPPL has at least one cleavage site located within the putative TMD of FBA. The cleavage sites outside TMD may be the result of stepwise cleavage by SPP/SPPLs or by other nonspecific aminopeptidase cleavage following SPP/SPPLs cleavage.

**Table 1:** Molecular weight of FLAG-BRI2 peptide detected in FBA/SPP co-transfected cell lysates.

Sequence	MW Calculated (Da)	MW Observed (Da)		
		SPPL2b	hSPP	pSPP
FLAG-MVKV…GQRRAWCWCMCFGLAFMLAGV	8524	8523 (-1)		
FLAG-MVKV…GQRRAWCWCMCFG	7720		7762(+42)*7803(+83)	7761 (+41)7804 (+84)
FLAG-MVKV…GQRRAWCWCMCF	7664		7663 (-1)7706(+42)	7663 (-1)7706 (+42)
FLAG-MVKV…GQRRAWCWCMC	7517		7558(+41)7601(+84)	7558 (+41)7602 (+85)
FLAG-MVKV…GQRRAWCWCM	7413		7455(+42)7497(+84)	7456 (+43)7497 (+84)
FLAG-MVKV…GQRRAWCWC	7282			7325 (+43)7366 (+84)

*The number in the brackets is the difference between observed and calculated MW. Error between observed and calculated mass is less than ±0.1%.

**Table 2:** Molecular weight of x-Aβ_1-25/K16A_ peptide detected in FBA/SPP co-transfected cell media.

Sequence	MW Calculated (Da)	MW Observed (Da)^a^		
		SPPL2b	hSPP	pSPP
GVILGGAYLYKYFAL-Aβ_1-25/K16A_	4506			4506
YLYKYFAL-Aβ_1-25/K16A_	3938		3939	3937
LYKYFAL-Aβ_1-25/K16A_	3775	3775		
KYFAL-Aβ_1-25/K16A_	3499	3500	3499	3499
YFAL-Aβ_1-25/K16A_	3371	3370	3371	3370
AL-Aβ_1-25/K16A_	3207		3207	3206
AL-Aβ_1-25/K16A_	3060	3060	3060	3060

^a^ Error between observed and calculated mass is less than ±0.1%.

### Established GSI Selectively inhibit SPPLs

**Fig 3:**
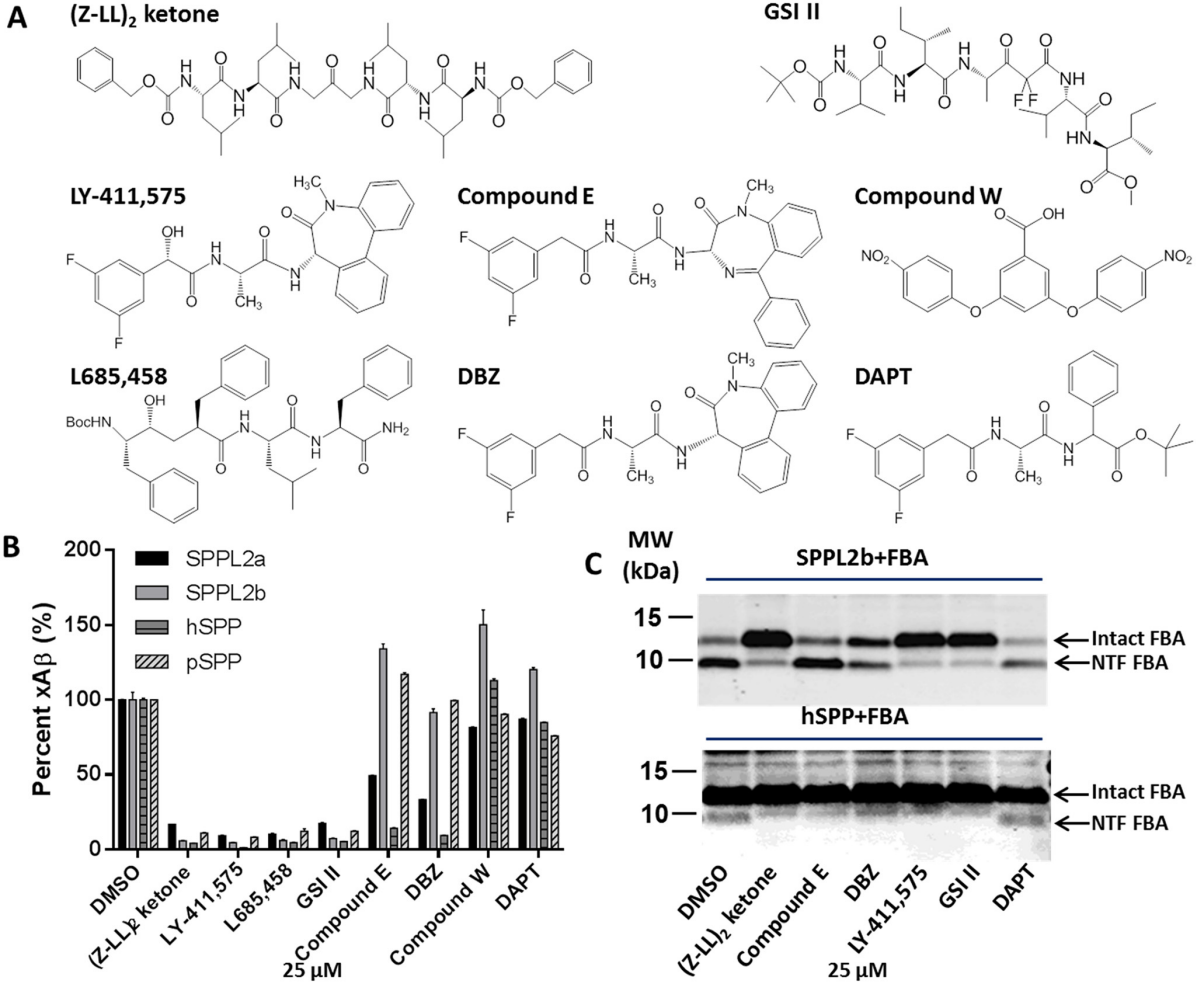
GSIs selectively inhibit SPP/SPPLs cleavage. A. Chemical structure of select GSIs. B. xAβ_1-25/K16A_ secretion from SPPLs co-transfected cell are largely inhibited by (Z-LL)_2_ ketone, LY-411,575, L685,458 and GSI II. The activity of hSPP was also inhibited by Compound E and DBZ. The xAβ_1-25/K16A_ concentrations in DMSO treated cultures are set as 100%. Statistical analysis performed by 1-way ANOVA ((*p<0.05, **p<0.01, ***p<0.001, ****p<0.0001). C. Western blot of FBA/SPPL2b (top) and FBA/hSPP (bottom) co-transfected cell with select GSIs.

In addition to (Z-LL)_2_ ketone, various GSIs have been used in the study of SPP/SPPLs, though these studies have not been systematic in nature. Using the ELISA method described above, we treated transiently co-transfected cells (FBA with each SPP/SPPL) with 25 μM of a subset of GSIs and (Z-LL)_2_ ketone (Fig 3A). (Z-LL)_2_ ketone, LY-411,575 and L685,458 show >90% inhibition of the SPP/SPPLs. GSI II also efficiently inhibited SPP/SPPLs without selectivity, whereas Comp W does not show inhibition of any SPP/SPPLs (Fig 3B). Notably, we found Comp E and DBZ selectively inhibit hSPP. To our knowledge, this is the first time that GSIs have been shown to selectively inhibit human SPP family members and show selectivity between a human SPP and the non-human pSPP. Western blotting further verified the selectivity of inhibition (Fig 3C). The upper panel shows that (Z-LL)_2_ ketone, LY-411,575 and GSI II greatly inhibit the cleavage conducted by SPPL2b. DBZ and DAPT slightly reduce the 10 kD ICD, while Comp E has negligible effect on FBA cleavage by SPPL2b. The lower panel shows that (Z-LL)_2_ ketone, LY-411,575, GSI II, Comp E and DBZ all efficiently inhibited hSPP cleavage.

**Fig 4:**
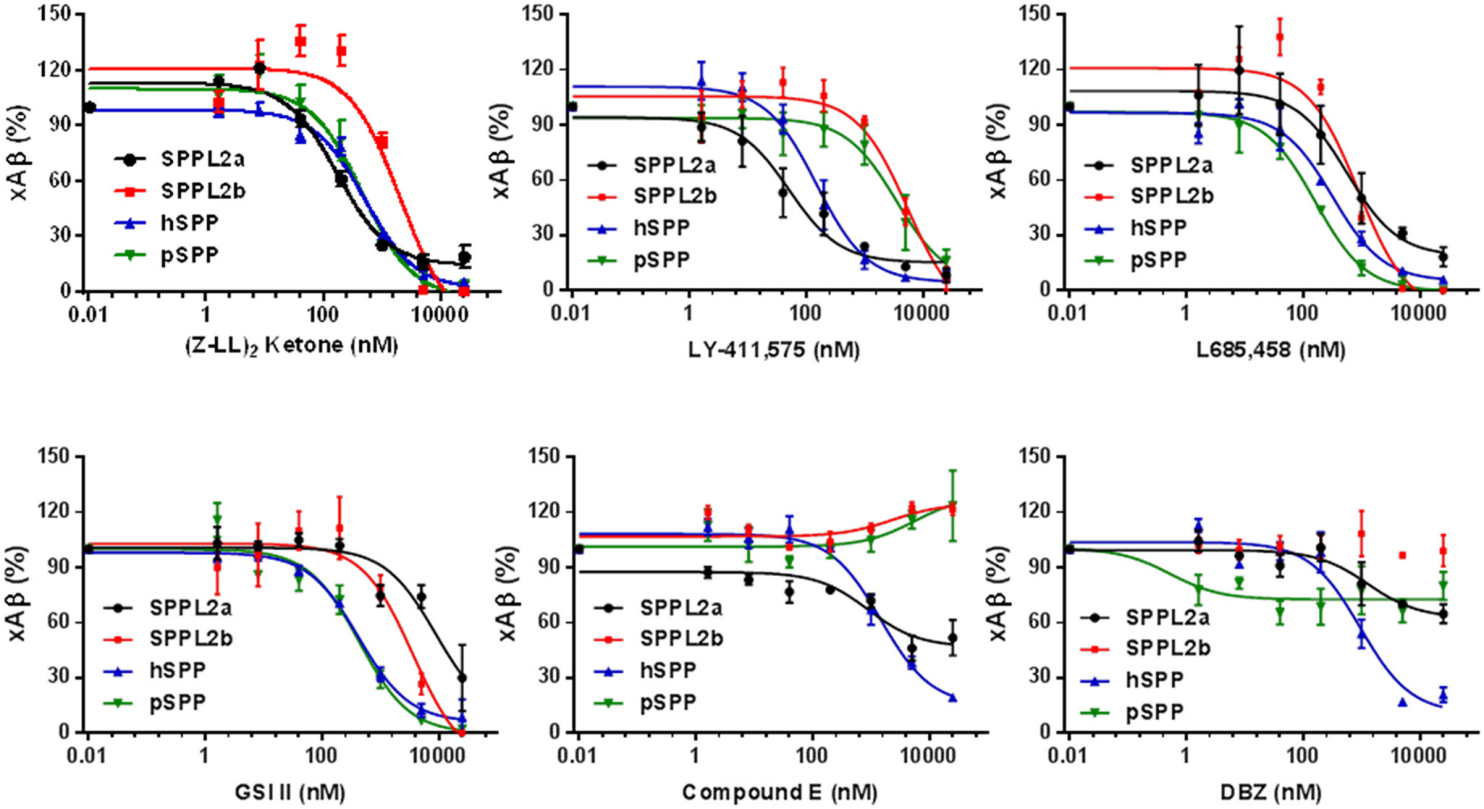
GSIs inhibit SPP/SPPLs in a dose-dependent manner. FBA/SPPLs co-transfected HEK cells were treated with DMSO or GSIs as appropriate. Twenty-four hours later the medium was collected for assay by Aβ ELISA. xAβ_1-25/K16A_ levels from DMSO-treated cells served as the control. All tests were repeated 3 times. Data were analyzed using GraphPad.

ELISA provides an efficient and quantitative means to perform a dose response study. Fig 4 and Table 3 summarize the dose response of (Z-LL)_2_ ketone and 5 GSIs on each SPP/SPPL. (Z-LL)_2_ ketone and L685,458 show modest selectivity on SPP/SPPLs. IC_50_s of (Z-LL)_2_ ketone varied from 177±99 to 2141±143 nM. IC_50_s of L685,458 varied from 161±97 to 876±133 nM. LY-411,575 and GSI II show greater selectivity on certain SPPLs. For example, LY-411,575 IC_50_ on SPPL2a and SPPL2 are 51±79 and 5499±122 nM respectively. The IC_50_ of GSI II on hSPP and SPPL2a are 423±92 and 8802±94 nM. Comp E did not show inhibition on SPPL2b and pSPP even at a relatively high concentration (25 μM), but it did inhibit SPPL2a slightly, though it did not reach 50% inhibition at the doses tested. Comp E however, efficiently inhibited hSPP with an IC_50_ of 1465±93 nM. The dose responses of DBZ on each SPPLs were similar to Comp E, showing only significant inhibition on hSPP with an IC_50_ of 948±93 nM. Western blot of the lysates from cells co-transfected with SPPL2b/FBA and treated with GSIs verify the dose response observed in ELISA (Panel C in S1 Fig).

**Table 3:** IC_50_s (nM) of select GSI on FBA/SPPL co-transfected cells.

	(Z-LL)_2_ ketone	LY-411,575	L685,458	GSI II	Compound E	DBZ
**SPPL2a**	177±99 (2)	51±79 (1)	570±90 (3)	8802±94 (4)	-	-
**SPPL2b**	2141±143 (2)	5499±122 (4)	876±133 (1)	3604±121(3)	-	-
**hSPP**	519±97 (4)	150±106 (1)	319±91(2)	423±92 (3)	1465±93	948±93
**pSPP**	472±114 (3)	3479±90 (4)	161±97 (1)	427±100 (2)	-	-

Each assay was repeated at least 3 times. The values in the table are the means ± standard errors. Numbers in parenthesis represent the rank order of inhibition of each SPP/SPPL by each compound.

**Fig 5:**
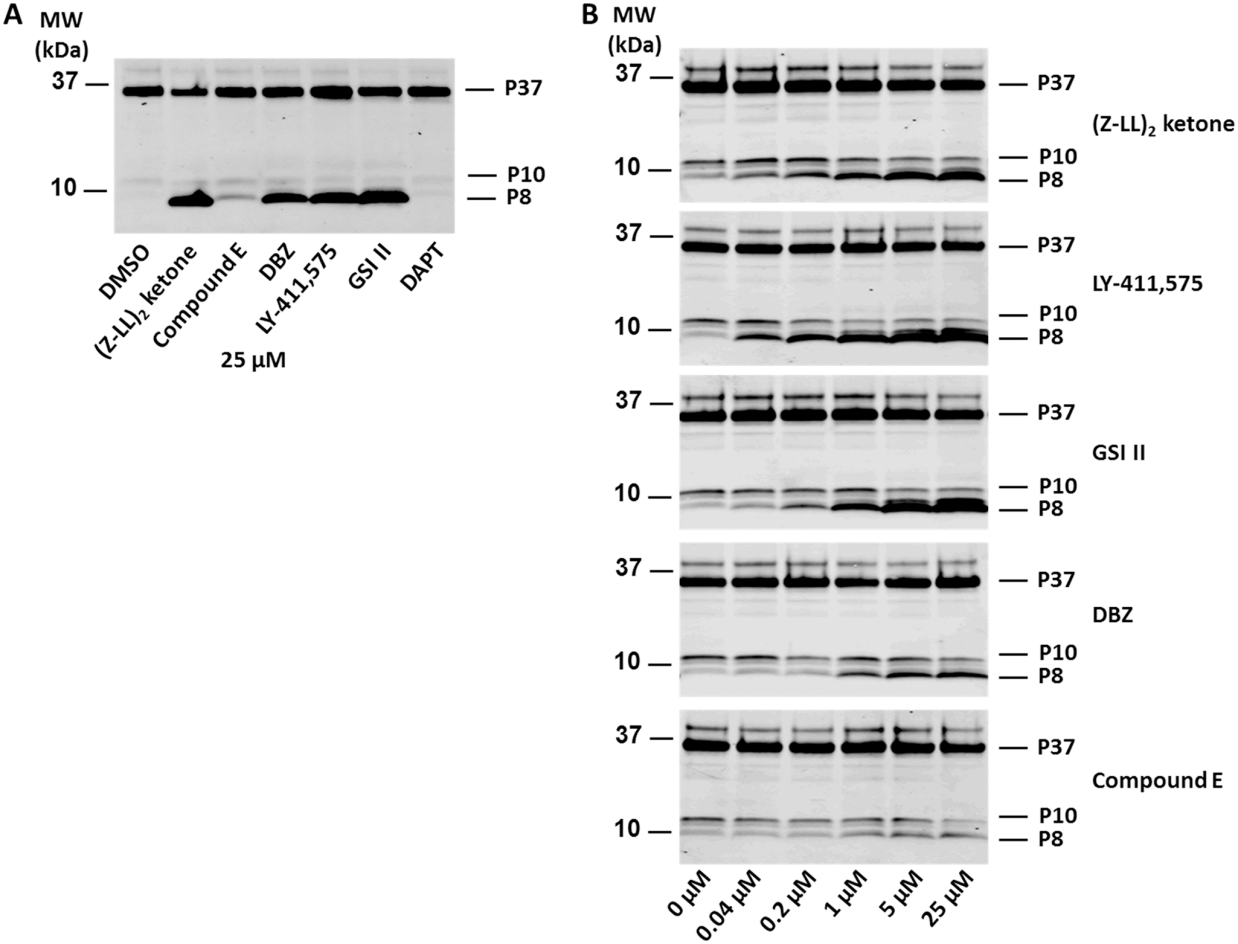
The selectivity of GSIs on SPPL is verified using non-overexpressing A20 cells. A. Western blot of A20 cells treated with 25 μM (Z-LL)_2_ ketone or GSIs. Blot was developed using anti-CD74 antibody In-1. P37 is the intact CD74. P10 and P8 are the 10 kD and 8 kD CD74 NTFs. Accumulation of P8 indicates SPPL2a inhibition. B. Dose response of A20 cell to (Z-LL)_2_ ketone, LY-411,575, GSI II, DBZ and Compound E.

To confirm the dose dependency of the GSIs on SPP/SPPLs, we used the mouse B lymphocyte, non-overexpressing cell line A20 (ATCC TIB-208). By analyzing the endogenous CD74 using In-1 antibody, we found 25 μM (Z-LL)_2_ ketone, LY-411,575 and GSI II greatly stabilized the CD74 8 kDa NTF P8 (Fig 5A) due to the impaired turnover of NTF [22]. DAPT showed no inhibition on this process at the given dose. Furthermore, we tested the inhibitors to A20 cells at different doses (Fig 5B). (Z-LL)_2_ ketone, LY-411,575, GSI II and DBZ stabilized the P8 fragment in a dose dependent manner. (Z-LL)_2_ ketone, LY-411,575, GSI II actively inhibited P8 turnover as low as 40 nM, whereas DBZ showed much weaker inhibition. The effect of DBZ was first seen at 1 μM. Comp E did not show significant effects even at 25 μM. Overall, these data from A20 cells show that findings from our co-transfections studies are largely confirmed in a more physiologic setting. Slight discrepancies between the dose response in our co-transfection studies and the studies A20 cells may be attributable to differences between mouse and human SPPL2a and the fact that other factors may influence the stability of the CD74 P8 NTF.

## Discussion

We have established a rapid cell-based assay platform for detecting proteolytic activity of SPP/SPPLs using the FBA substrate. When overexpressed in HEK cells hSPP, pSPP, SPPL2a and SPPL2b cleave FBA at sufficient levels to enable reliable detection of cleavage products. The artificial ectodomain of FBA containing the truncated BRI2 TMD and Aβ_1–25_ (xAβ_1–25_) can be sensitively and cost-effectively evaluated using an Aβ sandwich-ELISA. Significant cleavage was not detected by ELISA or Western blotting when only FBA was transfected, suggesting that i) endogenous levels of SPPs/SPPLs in HEK 293T cells are not sufficient to process the substrate or ii) that endogenous SPP/SPPLs are not co-localized with substrate at sufficient levels to detect cleavage. In the presence of transfected SPP/SPPLs, FBA was cleaved sufficiently, yielding xAβ_1–25_ levels in conditioned media well above background and within the dynamic range of our ELISA (5–3000 pM). Given the low background, sufficient target level, quantified detection, and rapid protocol, this assay could easily be adapted to a high throughput screening platform. As a single substrate and detection method is used to monitor cleavage of multiple SPP/SPPLs, this method may offer advantages over other screens previously reported including reporter assays previously developed in our laboratory[36].

In addition to processing of typical NH_2_-terminal signal peptides, SPP cleaves the internal membrane-spanning sequence of certain proteins, such as the immunoglobulin-like protein IgSF1 [46] and HCV core proteins [47]. Though a previous study suggests SPP does not cleave BRI2 protein[20], given that γ-secretase cleaves numerous transmembrane substrates irrespective of primary sequence, as well as prior ectodomain shedding of the substrate as key regulators of SPP/SPPL cleavage, it is not surprising that hSPP and pSPP also cleave FBA efficiently when substrate and protease are overexpressed. Substrate/enzyme overexpression and mislocalization may account for the differences in observed between cleavage in cells and our FBA substrate. However, MALDI-TOF-MS analysis demonstrates that SPPL2b, hSPP and pSPP have different preferential cleavage sites within the FBA substrate. Analysis of the hSPP and pSPP processed ICDs show that most of the products are either mono- or di-acetylated. Substrate acetylation has previously been reported inSPPL2b mediated TNF processing [18]. Our study, along with one from Fluhrer and colleagues, utilized substrates fused with DYKDDDDK (FLAG) tag, and it remains unclear whether the acetylation is on the lysine residues of the tag or on the actual substrates. As there is some evidence that acetylation may play a role in ER-associated degradation (ERAD) of proteins [48–50], further investigation of SPP/SPPL substrate acetylation is needed to determine the role acetylation plays in regulating SPP/SPPL mediated cleavage. IP/MS of conditioned media show each SPP/SPPL has at least one secreted product (xAβ_1–25_) that starts within the putative TMD, but additional SPP/SPPL dependent cleavages that extend into the ectodomain exist.

Our data suggest that hSPP, pSPP and SPPL2b processing of FBA results in i) a gap between the carboxyl end of the ICD and the NH_2_-terminus of the CTF, a finding noted previously for SPPL2b cleavage of TNFα [18], ii) multiple ICD generation by hSPP and pSPP and iii) multiple CTF generation by all three enzymes. Processing of substrates by SPP/SPPLs is highly reminiscent of γ-secretase substrate processing, with the exception that SPP/SPPLs the process is inverted in terms ICDs and CTFs [51–53]. γ-Secretase appears to initially cleave the TMD of substrate at a site or sites proximal to the cytoplasmic face of the membrane releasing a CTF into the cytoplasm and a membrane associated NTF. Following this initial cleavage,γ-secretase then catalyzes a series of step-wise cleavages on the NTF that result in the secretion of peptides that are typically 5–12 amino acids shorter than the initial fragment produced. As all characterized cleavage products of the FBA substrate can be inhibited by treatment with SPP inhibitors, our current data reinforce the previous studies which also suggest that SPP/SPPL cleavage may occur in several steps: an initial cleavage or cleavage(s) presumably generating ICD(s) released into the cytoplasm and subsequent processive cleavages that generate multiple CTF(s) that may be secreted in certain circumstances. Proving such a mechanism will likely require additional *in vitro* studies that enable detection of the step-wise cleavage products such as those conducted on γ-secretase by Ihara colleagues[51]. In an in vitro SPP-catalyzed cleavage assay based on synthetic prolactin signal peptide, the majority of the cleavage occurred at a single site[43], thus it is possible that the heterogeneous cleavages observed can be attributed to initial SPP/SPPL catalyzed cleavage with additional trimming by other proteases.

Previous studies of SPP/SPPLs have demonstrated that cleavage occurs following ectodomain shedding by signal peptidase (SP) for hSPP and by ADAM family members for SPPL2a/b[4, 54]. The FBA substrate used here is cleaved without prior ectodomain shedding, as the intact COOH-terminus is released into the media. For inhibitor screening, this observation is valuable, as the ELISA would not detect false-positive hits due to inhibition of various sheddases. These data also suggest that like γ-secretase, there is some tolerance for SPP/SPPL mediated cleavage of transmembrane substrates with ectodomains of lengths up to 30 amino acids. This later finding is somewhat unexpected for SPP because published studies that have mapped the carboxyl terminus of SPP substrate have only reported cleavage of substrates with ectodomains of six amino acids or less[43, 54]. In contrast SPPLs appear to cleave substrates with longer carboxyl termini. SPPL2b cleaves transferrin receptor-1, which has a predicted carboxyl terminal ectodomain of 12 amino acid residues[24, 55, 56] and SPPL2a cleaves the Fas ligand which has a predicted carboxyl terminal ectodomain of 20 amino acid[57].

Two of the GSIs LY-411,575 and L685,458 tested here have been shown to be inhibitors of SPP/SPPLs; however, the GSI DAPT, which is structurally related to LY-411,575, does not inhibit SPP/SPPLs [36–38]. Both of these are findings we confirm here. Curiously, (Z-LL)_2_ ketone, a peptide-based inhibitor designed to mimic the leucine-rich hydrophobic core of signal peptides cleaved by SPP, was originally reported as a selective inhibitor SPPs/SPPLs, but we have found it acts as an iGSMs on γ-secretase; thus, it clearly interacts with γ-secretase [39, 40]. Similarly, the GSM sulindac sulfide, was also reported to shift the main cleavage product of synthetic prolactin signal peptide (prl) generated by hSPP *in vitro* [43]. In this work we found that the GSI, GSI II, inhibits both SPP/SPPLs and PSs at sub-μM to μM doses [58]. Finally, we would note that SPPL2b cleavage of FBA is increased by low concentrations of several inhibitors (Fig 3), a phenomenon also observed under some circumstances in studies of γ-secretase cleavage of APP [59]. Collectively these data further reinforce the functional similarities and differences among SPPs/SPPLs and PSENs/γ-secretase in their proteolytic mechanisms.

Based on known functions of SPPs/SPPLs, it is likely that an inhibitor common to all SPPs/SPPLs would have side effects. Our data demonstrate that developing selective inhibitors is in principle, feasible; however, development of selective inhibitors will likely require extensive iterative structure-activity relationship (SAR) studies. From the limited SAR study conducted here, we can garner that the hydroxyl group present on LY-411,575 and absent on DBZ (Fig 3), may be a critical determinant that enhances inhibition of SPP/SPPLs. The lack of this hydroxyl group may also contribute to the lack of inhibition of SPP/SPPLs by DAPT. More generally, we can clearly show that different GSIs have selective effects on the SPP/SPPLs. For example, two GSIs, Compound E and DBZ, selectively inhibit hSPP cleavage. These two inhibitors differentiate, not only between the two subgroups (SPPs and SPPLs), but also among SPPs from different organisms (human and plasmodium). One potential caveat to our data, which may also explain differences in IC_50_s for hSPP by (Z-LL)_2_ ketone and the IC_50_s originally reported [2, 36], is the high level of SPP/SPPL present in the cell upon overexpression resulting in the artificial increase of IC_50_s. Thus, we believe a conservative approach to interpretation of our IC_50_ data (Fig 3 and Table 3) is to compare the rank order of inhibition for each protease by each inhibitor, as the relative IC_50_ will not be influenced by level of the protein. Even using this conservative approach to the data, it is clear that the compounds differentially inhibit the various SPP/SPPLs. Though the inhibition profile presented here may not reflect ideal physiological conditions, as BRI2 might not be a natural substrate of hSPP and pSPP[20], the assay system we reported here does provide rapid drug-enzyme interaction information.

The data presented here indicates the promise, as well as the challenges, in the development of specific inhibitors as potential SPP/SPPL targeting therapeutics for protozoan diseases such as malaria [15, 37, 60–62]. There is very strong evidence that inhibition of pSPP kills *Plasmodium* parasites [60]. Considering that *Plasmodium* parasites have only one copy of SPP, a pSPP specific inhibitor could potentially treat malaria without affecting the physiological function of hSPP/SPPLs. Further, recent work from SPPL2a deficient mice suggests that SPPL2a may be a target for immunomodulatory therapies and possibly in B-cell neoplasms [21–23, 25]. It is hoped that in future studies, we can use the screening platform for SPPs/SPPLs developed herein to identify and optimize selective inhibitors of these proteases. Indeed, the data here indicate that current inhibitors originally developed as inhibitors of γ-secretase, differentially inhibit various SPP family members. In addition, to our knowledge, current GSIs now repurposed in clinical trials for various cancers have not been systematically evaluated for inhibition of SPP/SPPLs [40]. Screening those clinical candidates for crosstalk with SPP/SPPL2 may reveal important insights into their potential efficiency and side effect profile.

## Supporting Information

S1 Fig A. FBA/pSPP co-transfect cell lysate IP/MS. Peaks at 7325 Da and 7805 Da match calculated molecular weight of single acetylated FLAG-BRI21-C56 and double acetylated FLAG-BRI21-G60. B. Western blot of FBA detected with anti-acetylated lysine antibody 15G10 (Biolegend, San Diego, CA, USA). C. Western blot of FBA/SPPL2b co-transfect cell lysate. Cells were treated with (Z-LL)_2_ ketone, LY-411,575, GSI II and Compound E at given concentration.
(TIF)
