# Supplementary figures and images for: Differential Inhibition of Signal Peptide Peptidase Family Members by Established γ-Secretase Inhibitors
Source: PLoS One. 2015 Jun 5;10(6):e0128619. doi: 10.1371/journal.pone.0128619 (PMC4457840; doi:10.1371/journal.pone.0128619)

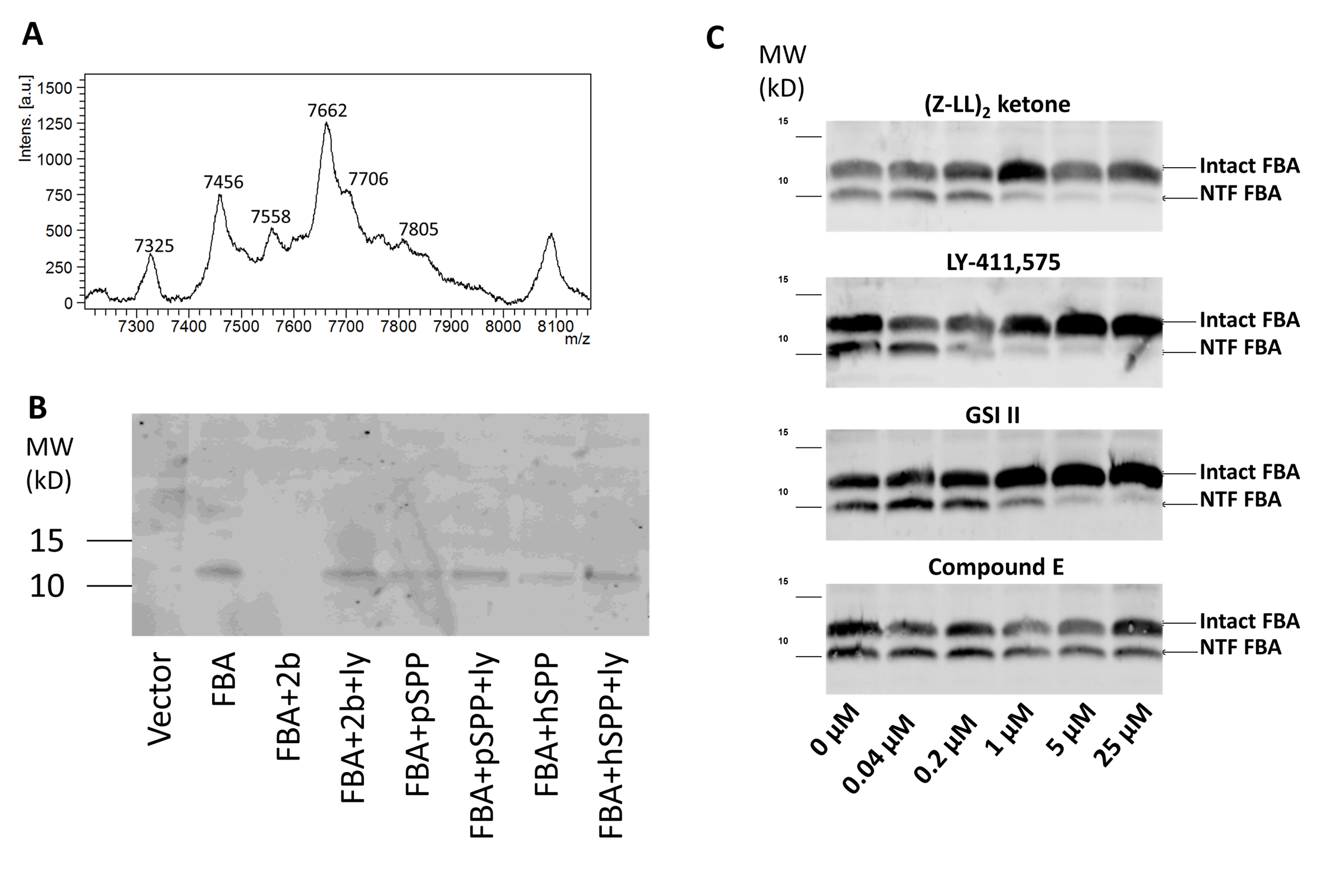

Supplement: S1 Fig — A. FBA/pSPP co-transfect cell lysate IP/MS. Peaks at 7325 Da and 7805 Da match calculated molecular weight of single acetylated FLAG-BRI21-C56 and double acetylated FLAG-BRI21-G60. B. Western blot of FBA detected with anti-acetylated lysine antibody 15G10 (Biolegend, San Diego, CA, USA). C. Western blot of FBA/SPPL2b co-transfect cell lysate. Cells were treated with (Z-LL)2 ketone, LY-411,575, GSI II and Compound E at given concentration. (TIF) [file pone.0128619.s001.tif]
